# Hyperkalemia Causing Inappropriate Subcutaneous Implantable Cardioverter Defibrillator Shocks in a Patient with End-Stage Renal Disease: A Case Report and Literature Review

**DOI:** 10.7759/cureus.31137

**Published:** 2022-11-05

**Authors:** Bachar Botrus, Arun U Mahtani, Ryan Isber, Muhammad S Haider, Nidal Isber

**Affiliations:** 1 Internal Medicine, Richmond University Medical Center, New York, USA; 2 Biology, Binghamton University, Binghamton, USA; 3 Electrophysiology, Richmond University Medical Center, New York, USA

**Keywords:** end stage renal disease (esrd), subcutaneous implantable cardioverter, implantable-cardioverter defibrillator, defibrillator shock, shock, electrodes, defibrillators, hyperkalemia, chronic renal insufficiency

## Abstract

Subcutaneous implantable cardioverter-defibrillators (S-ICD) provide an effective treatment option for ventricular arrhythmias. When compared to transvenous implantable cardioverter-defibrillators (TV-ICDs), S-ICDs have a lower infection rate but a higher rate of inappropriate shocks. In patients with end-stage renal disease (ESRD), significant electrolyte disturbances are commonly seen, such as hyperkalemia, which can cause an increase in T wave amplitude. We present a patient with ESRD on hemodialysis who experienced inappropriate shocks from an S-ICD during sinus rhythm due to hyperkalemia-induced T wave oversensing and highlight related cases in the current literature.

## Introduction

Implantable cardioverter-defibrillators (ICD) are effective for the primary and secondary prevention of sudden cardiac death [1]. However, implantation of endocardial leads is associated with significant adverse events. Subcutaneous implantable cardioverter-defibrillators (S-ICD) are an alternative treatment option for ventricular arrhythmias [2]. It eliminates complications associated with traditional transvenous ICDs (TV-ICDs) [3]. Due to subcutaneous electrodes, rhythm detection and discrimination are challenging. T wave oversensing (TWOS) remains the main culprit for causing inappropriate shocks [4].

## Case presentation

A 60-year-old woman with a medical history of end-stage renal disease (ESRD) on hemodialysis secondary to polycystic kidney disease since the age of 35 (Monday, Wednesday, and Friday) presented to the emergency department due to shortness of breath, chest pain, and two unprovoked shocks from her S-ICD for the first time since placement. She presented with good compliance, anemia (baseline hemoglobin: 9-10 g/dL), hypertension, asthma, HIV on highly active antiretroviral therapy (HAART), heart failure with reduced ejection fraction (HFrEF; with an EF of 23%) due to nonischemic cardiomyopathy, and an S-ICD placed in the posterior axillary line with the subcutaneous electrodes traversing the right sternal border (Boston Scientific Emblem-MRI S-ICD A219/201798) for primary prevention in 2016. She was at a party drinking hard liquor when she received her first shock. Twenty-five minutes later, she received a second one. In addition, she complained of a mildly productive cough but denied fever, chest pain, paroxysmal nocturnal dyspnea, orthopnea, nausea, vomiting, or worsening of lower extremity edema. She admitted to eating bananas and potatoes frequently.

**Figure 1:**
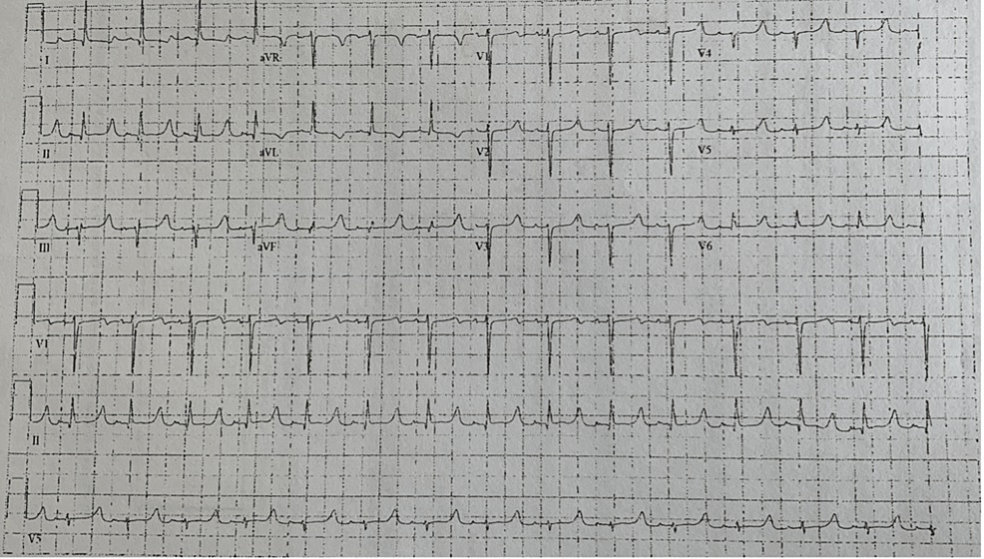
The 12-lead EKG shows normal sinus rhythm at a rate of 89 beats per minute with poor R wave progression in the anterior leads, peaked T waves, and a prolonged QTc interval of 537 ms.

Laboratory results showed a hemoglobin of 11.9 g/dL, sodium of 138 mmol/L, potassium of 7.4 mmol/L, creatinine of 6.6 mg/dL, a troponin level of < 0.01 ng/dL, and a BNP of 2171 pg/mL. An electrocardiogram (EKG) showed a normal sinus rhythm at a rate of 89 beats per minute (bpm) with poor R wave progression in the anterior leads, peaked T waves, and a prolonged QTc interval of 537 ms (Figure 1).

The chest x-ray showed no consolidation, effusion, or pneumothorax.

**Figure 2:**
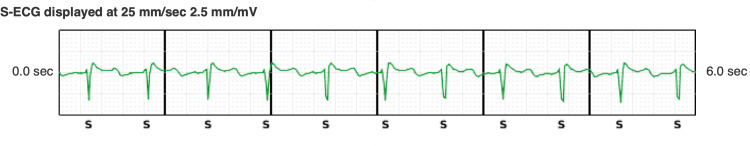
Surface electrocardiogram from the S-ICD: high QRS amplitude and appropriate QRS-T complex discrimination with normal potassium levels

During the patient’s most recent assessment in the device clinic, her presenting surface electrocardiogram (S-ECG) from the S-ICD showed a good amplitude of the QRS complex and appropriate QRS-T complex discrimination without T wave over-sensing or therapies (appropriate or inappropriate). This is shown in Figure 2.

Her S-ICD had been programmed to a shock zone of 240 bpm and a conditional shock zone of 200 bpm with post-shock pacing turned on and SMART Pass turned off.

Following the shocks, device interrogation showed two episodes of tachycardia detected, with two shocks delivered during sinus rhythm, even though it was well below the programmed tachycardia zones. Double-counting due to T wave oversensing led to tachycardia detection and the delivery of a shock.

**Figure 3:**
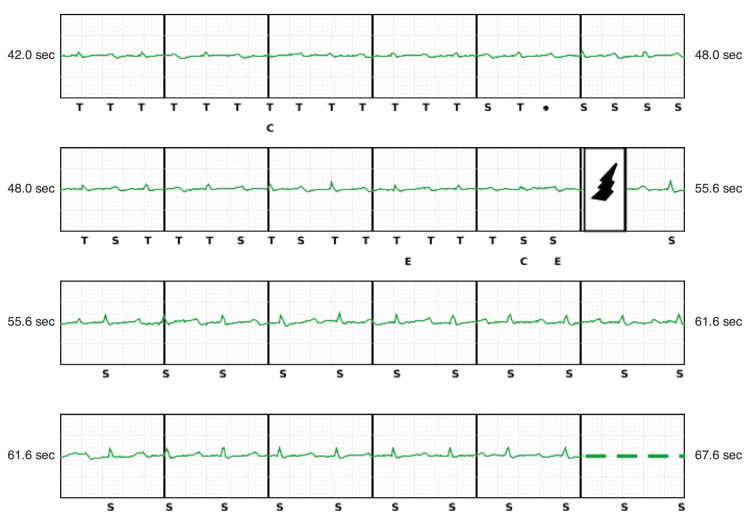
Inappropriate S-ICD shock during sinus rhythm with a rate of 100 bpm. Note low-amplitude QRS complexes relative to T waves with consistent oversensing resulting in double counting and satisfying tachycardia detection with subsequent delivery of an S-ICD shock (bolt).

The first episode demonstrates sinus rhythm at nearly 100 bpm with very low QRS amplitude and a tall T wave amplitude. There is appropriate QRS complex sensing and consistent T wave oversensing, resulting in double counting and a rate falling in the tachycardia detection zone, triggering a shock that resulted in the temporary disappearance of T wave oversensing (Figure 3).

**Figure 4:**
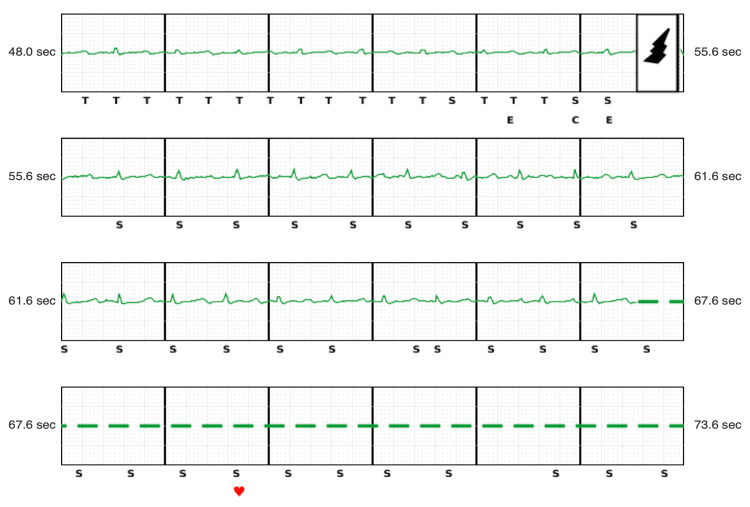
Second shock during sinus rhythm.

A second episode occurred about 15 minutes later (Figure 4), which showed sinus rhythm also around 100 bpm with QRS sensing and consistent T wave oversensing, resulting in double counting and a rate falling in the tachycardia detection zone, triggering a shock. No T wave oversensing was observed after the second shock.

Following interrogation of the device on admission, the conditional shock zone was reprogrammed from 200 bpm to 220 bpm, and the SMART Pass algorithm was switched on. The sensing configuration was changed from secondary to alternate.

She underwent urgent hemodialysis for two hours, and 1.5 liters of ultrafiltrate (UF) were removed using an Opti 160 dialyzer with a 1K bath. A repeated basic metabolic panel (BMP) showed a serum creatinine level of 4.9 g/dL and a potassium level of 5.1 mmol/L.

On day two of admission, her basic metabolic panel (BMP) showed serum potassium of 5.8 mmol/L. She underwent another round of dialysis for three hours, and 2.5L of UF was removed. A final BMP showed serum potassium of 4.7 mmol/L. A repeat EKG showed a normal sinus rhythm with no peaking of T waves and a QTc interval of 477 ms. With no further episodes of shocks, she was discharged and was asked to follow up with her electrophysiologist on an outpatient basis.

## Discussion

S-ICDs are a less invasive alternative when compared to conventional transvenous ICD systems. It is preferred in young patients, those with poor vascular access, and those at high risk for bacteremia, such as patients receiving hemodialysis. Although S-ICDs are associated with lower infection rates, there is a higher rate of inappropriate shocks secondary to TWOS. The 360-day rate reported from the EFFORTLESS S-ICD registry is about 7% [5]. It can range from 12%-29%, with the most common cause of inappropriate shocks being signal oversensing [4,6]. Hyperkalemia decreases the amplitude of depolarization (QRS complexes) and increases the amplitude of repolarization (T waves), making the device vulnerable to TWOS. This leads to double counting of the QRS complexes, causing inappropriate shocks. Transient hyperkalemia, common in dialysis patients, represents one of the mechanisms leading to TWOS and inappropriate shocks.

Kiamanesh et al. reported the case of a 33-year-old man with S-ICD for dilated cardiomyopathy (EF: 25%) and end-stage renal disease on dialysis who was admitted to the hospital after syncope [7]. He missed a scheduled session of hemodialysis and had a serum potassium level of 7.0 mmol/L. Interrogation of his S-ICD showed five episodes of tachycardia and 17 shocks. Due to TWOS, a few shocks were delivered on the T wave, which induced non-sustained polymorphic ventricular tachycardia and ventricular fibrillation. It was converted to sinus rhythm after four shocks, and the patient received urgent dialysis. Following this episode, the conditional shock zone and shock zone were increased to 200 and 230 bpm, respectively.

Chua et al. reported the case of a 58-year-old man with an S-ICD for non-ischemic cardiomyopathy and end-stage renal disease on hemodialysis who was admitted for drowsiness, lethargy, and shocks from his S-ICD [8]. Laboratory tests showed severe hyperkalemia of 9.7 mmol/L. Interrogation of the S-ICD showed a broad ventricular escape rhythm, at which the device was triple counting the QRS, and T wave complexes causing the device to oversense and fall in the tachycardia detection range, leading to inappropriate shocks. He too missed a session of hemodialysis, and urgent dialysis was initiated for rapid correction of his hyperkalemia. A differentiating point in our case is the absence of missed hemodialysis sessions. The only cause of his electrolyte abnormality could be attributed to her consumption of foods rich in potassium, such as bananas. TWOS can also occur from postural changes. Afzal et al. reported a 38-year-old male with a past medical history of hypertrophic obstructive cardiomyopathy (HOCM) who received an S-ICD due to primary prevention. In this patient, postural changes lead to the diminishing of the amplitude of QRS complexes, leading to TWOS [9]. Another case of TWOS leading to position changes was reported by Al-Khatib et al in a 58-year-old male with a medical history of non-ischemic cardiomyopathy, ESRD on HD, hypertension, new-onset atrial fibrillation, and Type 2 diabetes mellitus. In this patient, there was positional attenuation of the R waves that led to an activation of the device algorithm to increase the amplitude of the cardiac signals, resulting in the oversensing of the atrial fibrillation waves [10]. Our patient did not have any history of HOCM, atrial fibrillation, or shocks related to changes in posture. Ochman et al. reported the case of a 55-year-old male with a past medical history of coronary artery disease (CAD) status post coronary artery bypass graft (CABG), heart failure with reduced ejection fraction (HFrEF), who underwent S-ICD placement due to multiple pocket revisions after TV-ICD placement [11]. On interrogation, it was found that the device oversensed both P and T waves. In our case, there was an oversensing of only T waves. Oranus et al. reported a case of TWOS due to a change in the electrical vector following T wave remodeling and progression of the hypertrophic obstructive cardiomyopathy (HOCM) scar, causing loss of R waves in a 19-year-old male with a history of HOCM and Wolff-Parkinson-White (WPW) syndrome [12]. A similar case of cardiac remodeling leading to uncorrectable T wave oversensing was reported by Saleem et al. [13]. However, in our patient, there was TWOS due to an increase in T wave amplitude following hyperkalemia, which was corrected after the patient received hemodialysis. Yang et al. reported a case of a 58-year-old female patient with a medical history of anthracycline-induced cardiomyopathy with a low ejection fraction who developed TWOS due to artifacts from air entrapment inside the patient [14]. Our patient did not have any air in her device's pocket. Sousa et al. reported inappropriate shocks due to a rate-related right bundle branch block [15]. Our patient did not have any rate-related EKG changes.

SMART Pass and conditional zones are algorithms developed to reduce the burden of inappropriate shocks [16]. A SMART Pass is an advanced algorithm within the EMBLEM MRI S-ICD system that filters out certain signals that cause inappropriate shocks while accurately detecting dangerous rhythms and delivering lifesaving therapy. The algorithm showed a 68% risk reduction for inappropriate shocks without impacting the delivery of appropriate shocks [17].

## Conclusions

Clinicians managing patients with ESRD who have S-ICDs should be aware of the risk of inappropriate S-ICD shocks due to TWOS. It can occur in electrolyte disturbances such as hyperkalemia, and early detection may prevent them. TWOS can be prevented by the correct placement of the device without any air entry, reprogramming of the device, changing thresholds, utilization of various algorithms built within the device, implementing higher shock thresholds, and adjusting the sensing vector.
